# Literary Notes

**Published:** 1905-10

**Authors:** 


### LITERARY NOTES.

D. O. HAYNES & Co., publishers *of The Pharmaceutical Era,* have issued the "Era Key to the U. S. Pharmacopeia" in vest-pocket size which contains the essential information pertaining to the eighth decennial revision. It will aid every physician who desires to prescribe official pharmaceutical remedies, as it gives names, synonyms, and constituent parts, with average doses both in metric and apothecaries' systems.

---

THE H. K. Mulford Company, manufacturing chemists, Philadelphia, has prepared a "change sheet" for the use of prescription druggists, which gives at a glance the important changes made in the eighth decennial revision of the U. S. Pharmacopeia. It is a most useful chart and should be posted behind every prescription counter. This house is preparing a small folder giving these changes, for physicians' use, which will be furnished on request by the Mulford Company.

